# Supplementary material for: Feasibility of Continuous Monitoring of Endoscopy Performance and Adverse Events: A Single-Center Experience
Source: Cancers (Basel). 2023 Jan 24;15(3):725. doi: 10.3390/cancers15030725 (PMC9913416; doi:10.3390/cancers15030725)
Supplement: Supplementary file 1 [file cancers-15-00725-s001.zip › Table S1 Severe AEs and risk profile of endoscopic procedures and patients.pdf]

Table S1. Severe AEs and risk profile of endoscopic procedures and patients.

Type of endoscopy	All severe AEs				Sedation-related				Endoscopy-related immediate				Endoscopy-related late			
	Total		ASA > II		Total		ASA > II		Total		ASA > II		Total		ASA > II	
	n	%	n	%	n	%	n	%	n	%	n	%	n	%	n	%
**Low-Risk**	3	14	3	14	1	5	1	5	0	0	0	0	2	10	2	10
**Gastroscopy diagn.**	1	4.8	1	4.8	1	4.8	1	4.8	0	0.0	0	0.0	0	0.0	0	0.0
**Colonoscopy diagn.**	2	9.5	2	9.5	0	0.0	0	0.0	0	0.0	0	0.0	2	9.5	2	9.5
**Sigmoidoscopy**	0	0.0	0	0.0	0	0.0	0	0.0	0	0.0	0	0.0	0	0.0	0	0.0
**EUS diagnostic**	0	0.0	0	0.0	0	0.0	0	0.0	0	0.0	0	0.0	0	0.0	0	0.0
**High-Risk**	18	86	6	29	2	10	0	0	6	29	2	10	11	52	4	19
**OGIT ther.**	3	14.3	2	9.5	0	0.0	0	0.0	0	0.0	0	0.0	3	14.3	2	9.5
**Of which: bougie**	0	0.0	0	0.0	0	0.0	0	0.0	0	0.0	0	0.0	0	0.0	0	0.0
**EVL**	2	9.5	2	9.5	0	0.0	0	0.0	0	0.0	0	0.0	2	9.5	2	9.5
**Gastroscopy EMR**	0	0.0	0	0.0	0	0.0	0	0.0	0	0.0	0	0.0	0	0.0	0	0.0
**ESD OGIT**	1	4.8	0	0.0	0	0.0	0	0.0	0	0.0	0	0.0	1	4.8	0	0.0
**PEG plant**	0	0.0	0	0.0	0	0.0	0	0.0	0	0.0	0	0.0	0	0.0	0	0.0
**BS OGIT**	0	0.0	0	0.0	0	0.0	0	0.0	0	0.0	0	0.0	0	0.0	0	0.0
**UGIT ther.**	7	33.3	3	14.3	0	0.0	0	0.0	4	19.0	1	4.8	4	19.0	2	9.5
**Of which: EMR**	3	14.3	1	4.8	0	0.0	0	0.0	1	4.8	0	0.0	2	9.5	1	4.8
**Colonoscopy ESD**	4	19.0	1	4.8	0	0.0	0	0.0	3	14.3	1	4.8	1	4.8	0	0.0
**BS UGIT**	0	0.0	0	0.0	0	0.0	0	0.0	0	0.0	0	0.0	0	0.0	0	0.0
**EUS with biopsy**	1	4.8	0	0.0	0	0.0	0	0.0	0	0.0	0	0.0	1	4.8	0	0.0
**ERCP**	7	33.3	1	4.8	2	9.5	0	0.0	2	9.5	1	4.8	3	14.3	0	0.0
**DBE**	0	0.0	0	0.0	0	0.0	0	0.0	0	0.0	0	0.0	0	0.0	0	0.0
**Total**	**21**	**100.0**	**9**	**42.9**	**3**	**14.3**	**1**	**4.8**	**6**	**28.6**	**2**	**9.5**	**12**	**57.1**	**6**	**28.6**

Abbreviations: ASA: American Society of Anesthesiologists classification, diagn.: diagnostic, EUS: endosonography, OGIT: upper gastrointestinal tract, ther.: therapeutic, EVL: endoscopic variceal ligation, EMR: endoscopic mucosal resection, PEG: percutaneous endoscopic gastrostomy, BS: hemostasis, UGIT: lower gastrointestinal tract, ERCP: endoscopic retrograde cholangiopancreaticography, DBE: double balloon enteroscopy.
