# Supplementary material for: Feasibility of Continuous Monitoring of Endoscopy Performance and Adverse Events: A Single-Center Experience
Source: Cancers (Basel). 2023 Jan 24;15(3):725. doi: 10.3390/cancers15030725 (PMC9913416; doi:10.3390/cancers15030725)
Supplement: Supplementary file 1 [file cancers-15-00725-s001.zip › Table S2 Sedation regimen and AEs.pdf]

Table S2. Sedation regimen and AEs: Risk profile of patients (univariate analysis).

Sedation regime	Sedation-related AEs																	
	All						Major						Minor					
	Total		ASA > II				Total		ASA > II				Total		ASA > II			
	n	%	n	%	p [1]	p [2]	n	%	n	%	p	p	n	%	n	%	p	p
**Propofol mono**	140	2.0	48	0.7	0.003	0.003	3	0.04	2	0.03	0.001	0.002	137	1.99	46	0.7	0.003	0,002
**Propofol / Mid.**	16	11.3	9	6.3	0.286	0.400	0	0.0	0	0.0	n.a.		16	11.27	9	6.3	0.286	0,400
**Mid. mono (allergy)**	0	0.0	0	0.0	n.a.		0	0.0	0	0.0	n.a.		0	0.00	0	0.0	n.a.	
**Mid. mono (ASA > II)**	4	12.5	4	12.5	n.a.		0	0.0	0	0.0	n.a. [3]		4	12.50	4	12.5	n.a.	
**Anesth. Emergency [4]**	2	14.3	0	0.0	n.a.		2	14.3	0	0.0	n.a.		n.a. [5]		n.a.		n.a.	
**Anesth. ITN elective**	0	0.0	0	0.0	n.a.		0	0.0	0	0.0	n.a.		n.a.		n.a.		n.a.	
**Anesth. sed. elective**	0	0.0	0	0.0	n.a.		0	0.0	0	0.0	n.a.		n.a.		n.a.		n.a.	
**Total**	162	2.3	61	0.9			5	0.1	2	0.03			157	2.2	59	0.8		

Abbreviations: ASA: American Society of Anesthesiologists classification, Mid.: Midazolam, n.a.: not possible, Anesth.: Anesthesia, ITN: Intubation anesthesia, Sed.: Sedation.

[1] Analysis of variance using Chi-square / Fisher exact test

[2] Analysis of variance using Chi-square / Cramer's V

[3] not calculated, because only patients with ASA > II are included in this category.

[4] incl. intubation anesthesia

[5] Minor sedation-related immediate AEs during anesthesia management were not recorded in the AEPM documentation
