# Supplementary material for: Feasibility of Continuous Monitoring of Endoscopy Performance and Adverse Events: A Single-Center Experience
Source: Cancers (Basel). 2023 Jan 24;15(3):725. doi: 10.3390/cancers15030725 (PMC9913416; doi:10.3390/cancers15030725)
Supplement: Supplementary file 1 [file cancers-15-00725-s001.zip › Table S3 EndAE_lit.pdf]

Table S3. Endoscopy-related complications. Comparison with data from the literature.

Type of endoscopy			All adverse events				Minor				Major				
			This study		Literature		This study		Literature		This study		Literature		
		n	n	%	%	Ref.	n	%	%	Ref.	n	%	%	Ref.	Type
Low-Risk	**Total**	6054	21	0.6			19	0.55			2	0.03	0.03	1)	Delayed bleeding
	**Gastroscopy**	3510	10	0.28	0.13 - 0.15 [1]	2)	10	0.28			0				Bleeding after biopsy
	**Colonoscopy**	1791	11	0.6			9	0.50	1.0 - 2.0 [2]	3)	2	0.11	0.01 - 0.1 [3]	4)	Delayed bleeding
High-Risk	**Total**	1478	29	2.0			13	0.88			16	1.08			
	**Dilatation**	25	0	0.0	0.02 - 2.0	5)	0				0				
	**Variceal ligation**	234	9	3.8			7	2.99			2	0.85	8.0	6)	Delayed bleeding
	**PEG**	27	0		16 - 70	7)	0				0				
	**EMR**	109	0	0.0	5.0	8)	0				0				
	**ESD OGIT**	16	1	6.3	5.3	9)	0				1	6.25	5.3	9)	Delayed bleeding
	**EUS-FNA**	67					0				1	1.49	2.0 - 3.0	10)	Pancreatitis
	**ERCP**	377	13	3.4	7.0	11)	8	2.12	5.20		3	0.8	1.70	12)	Delayed bleeding, perforation
	**ERCP**										2	0.5	0.3 - 0.5	13)	Pancreatitis
	**DBE**	15	0	0.0	1.2 - 1.6	14)	0				0				
	**Ther. colonosc.**	405					0				7	1.7			
	**Colonosc. EMR**	359	3	0.8			0				3	0.84	0.1 - 5.0	15)	Delayed bleeding, perforation [4]
	**Colonosc. ESD**	46	4	8.7			0				3	6.5	2.0-15	16)	Delayed bleeding, perforation [4]
	**Colonosc. ESD**										1	2.20	1.0	17)	Emergency surgery
	**Total**	7532	56	0.7			38	0.50			18	0.24			

Abbreviations: Ref.: reference, PEG: percutaneous endoscopic gastrostomy, EMR: endoscopic mucosal resection, ESD: endoscopic submucosal dissection, OGIT: upper gastrointestinal tract, EUS-FNA: endosonography with fine needle aspiration, ERCP: endoscopic retrograde cholangiopancreaticography, DBE: double balloon enteroscopy, ther.: therapeutic, colonosc.: colonoscopy.

[1] incl. sedation-related adverse events

[2] incl. polypectomy

[3] Hemorrhage and perforation, incl. polypectomy

[4] Without surgery

Literature references:

1) [1], 2) [2, 3], 3) [4], 4) [5], 5) [6, 7], 6) [8, 9], 7) [10, 11], 8) [12], 9) [13], 10) [14], 11) [15], 12) [16, 17], 13) [18], 14) [19], 15) [5, 20], 16) [21, 22], 17) [22]

1. Chirica, M., A. Champault, X. Dray, L. Sulpice, N. Munoz-Bongrand, E. Sarfati, and P. Cattan. "Esophageal Perforations." *J Visc Surg* 147, no. 3 (2010): e117-28.
2. Silvis, S. E., O. Nebel, G. Rogers, C. Sugawa, and P. Mandelstam. "Endoscopic Complications. Results of the 1974 American Society for Gastrointestinal Endoscopy Survey." *JAMA* 235, no. 9 (1976): 928-30.
3. Geraci, G., F. Pisello, G. Modica, F. Li Volsi, E. Arnone, and C. Sciume. "[Complications of Elective Esophago-Gastro-Duodenoscopy (Egds). Personal Experience and Literature Review]." *G Chir* 30, no. 11-12 (2009): 502-6.
4. Waye, J. D., B. S. Lewis, and S. Yessayan. "Colonoscopy: A Prospective Report of Complications." *J Clin Gastroenterol* 15, no. 4 (1992): 347-51.
5. Whitlock, E. P., J. S. Lin, E. Liles, T. L. Beil, and R. Fu. "Screening for Colorectal Cancer: A Targeted, Updated Systematic Review for the U.S. Preventive Services Task Force." *Ann Intern Med* 149, no. 9 (2008): 638-58.
6. Shemesh, E., and A. Czerniak. "Comparison between Savary-Gilliard and Balloon Dilatation of Benign Esophageal Strictures." *World J Surg* 14, no. 4 (1990): 518-21; discussion 21-2.
7. Saeed, Z. A., C. B. Winchester, P. S. Ferro, P. A. Michaletz, J. T. Schwartz, and D. Y. Graham. "Prospective Randomized Comparison of Polyvinyl Bougies and through-the-Scope Balloons for Dilation of Peptic Strictures of the Esophagus." *Gastrointest Endosc* 41, no. 3 (1995): 189-95.
8. Sharma, M., S. Singh, V. Desai, V. H. Shah, P. S. Kamath, M. H. Murad, and D. A. Simonetto. "Comparison of Therapies for Primary Prevention of Esophageal Variceal Bleeding: A Systematic Review and Network Meta-Analysis." *Hepatology* 69, no. 4 (2019): 1657-75.
9. Petrasch, F., J. Grothaus, J. Mossner, I. Schiefke, and A. Hoffmeister. "Differences in Bleeding Behavior after Endoscopic Band Ligation: A Retrospective Analysis." *BMC Gastroenterol* 10 (2010): 5.
10. Larson, D. E., D. D. Burton, K. W. Schroeder, and E. P. DiMagno. "Percutaneous Endoscopic Gastrostomy. Indications, Success, Complications, and Mortality in 314 Consecutive Patients." *Gastroenterology* 93, no. 1 (1987): 48-52.
11. Blomberg, J., J. Lagergren, L. Martin, F. Mattsson, and P. Lagergren. "Complications after Percutaneous Endoscopic Gastrostomy in a Prospective Study." *Scand J Gastroenterol* 47, no. 6 (2012): 737-42.
12. Okano, A., K. Hajiro, H. Takakuwa, A. Nishio, and M. Matsushita. "Predictors of Bleeding after Endoscopic Mucosal Resection of Gastric Tumors." *Gastrointest Endosc* 57, no. 6 (2003): 687-90.
13. Koh, R., K. Hirasawa, S. Yahara, H. Oka, K. Sugimori, M. Morimoto, K. Numata, A. Kokawa, T. Sasaki, A. Nozawa, M. Taguri, S. Morita, S. Maeda, and K. Tanaka. "Antithrombotic Drugs Are Risk Factors for Delayed Postoperative Bleeding after Endoscopic Submucosal Dissection for Gastric Neoplasms." *Gastrointest Endosc* 78, no. 3 (2013): 476-83.
14. Jung, J. G., J. K. Lee, K. H. Lee, K. T. Lee, Y. S. Woo, W. H. Paik, D. H. Park, S. S. Lee, D. W. Seo, S. K. Lee, and M. H. Kim. "Comparison of Endoscopic Retrograde Cholangiopancreatography with Papillary Biopsy and Endoscopic Ultrasound-Guided Pancreatic Biopsy in the Diagnosis of Autoimmune Pancreatitis." *Pancreatology* 15, no. 3 (2015): 259-64.
15. Andriulli, A., S. Loperfido, G. Napolitano, G. Niro, M. R. Valvano, F. Spirito, A. Pilotto, and R. Forlano. "Incidence Rates of Post-Ercp Complications: A Systematic Survey of Prospective Studies." *Am J Gastroenterol* 102, no. 8 (2007): 1781-8.
16. Williams, E. J., S. Taylor, P. Fairclough, A. Hamlyn, R. F. Logan, D. Martin, S. A. Riley, P. Veitch, M. L. Wilkinson, P. R. Williamson, and M. Lombard. "Risk Factors for Complication Following Ercp; Results of a Large-Scale, Prospective Multicenter Study." *Endoscopy* 39, no. 9 (2007): 793-801.

17. Wang, P., Z. S. Li, F. Liu, X. Ren, N. H. Lu, Z. N. Fan, Q. Huang, X. Zhang, L. P. He, W. S. Sun, Q. Zhao, R. H. Shi, Z. B. Tian, Y. Q. Li, W. Li, and F. C. Zhi. "Risk Factors for Ercp-Related Complications: A Prospective Multicenter Study." *Am J Gastroenterol* 104, no. 1 (2009): 31-40.
18. Kochar, B., V. S. Akshintala, E. Afghani, B. J. Elmunzer, K. J. Kim, A. M. Lennon, M. A. Khashab, A. N. Kalloo, and V. K. Singh. "Incidence, Severity, and Mortality of Post-Ercp Pancreatitis: A Systematic Review by Using Randomized, Controlled Trials." *Gastrointest Endosc* 81, no. 1 (2015): 143-49 e9.
19. Moschler, O., A. May, M. K. Muller, C. Ell, and D. B. E. Study Group German. "Complications in and Performance of Double-Balloon Enteroscopy (Dbe): Results from a Large Prospective Dbe Database in Germany." *Endoscopy* 43, no. 6 (2011): 484-9.
20. Chukmaitov, A., C. J. Bradley, B. Dahman, U. Siangphoe, J. L. Warren, and C. N. Klabunde. "Association of Polypectomy Techniques, Endoscopist Volume, and Facility Type with Colonoscopy Complications." *Gastrointest Endosc* 77, no. 3 (2013): 436-46.
21. Stock, C., P. Ihle, A. Sieg, I. Schubert, M. Hoffmeister, and H. Brenner. "Adverse Events Requiring Hospitalization within 30 Days after Outpatient Screening and Nonscreening Colonoscopies." *Gastrointest Endosc* 77, no. 3 (2013): 419-29.
22. Wagner, A., D. Neureiter, T. Kiesslich, G. W. Wolkersdorfer, T. Pleininger, C. Mayr, C. Dienhart, N. Yahagi, T. Oyama, and F. Berr. "Single-Center Implementation of Endoscopic Submucosal Dissection (Esd) in the Colorectum: Low Recurrence Rate after Intention-to-Treat Esd." *Dig Endosc* 30, no. 3 (2018): 354-63.
