# Supplementary material for: Feasibility of Continuous Monitoring of Endoscopy Performance and Adverse Events: A Single-Center Experience
Source: Cancers (Basel). 2023 Jan 24;15(3):725. doi: 10.3390/cancers15030725 (PMC9913416; doi:10.3390/cancers15030725)
Supplement: Supplementary file 1 [file cancers-15-00725-s001.zip › Table S4 SedAE_lit.pdf]

Table S4. Sedation-related adverse events. Comparison with data from the literature.

Adverse events	This study			Literature [1]	
**Type**	n	% of all procedures	% of minor AE	%	% of minor AE
**Minor**	170	2.26	100.00	0.3	100.0
**Respiratory**	118	1.57	69.41		33.0
**Saturation drop**	114	1.51	67.06		33.0
**Aspiration**	6	0.08	3.53		7.0
**Apnea**	0	0.00	0.00		
**Laryngeal edema**	1	0.01	0.59		2.0
**Cardiovascular**	52	0.69	30.59		34.0
**Hypotension**	28	0.37	16.47		12.0
**Tachycardia**	7	0.09	4.12		10.0
**Bradycardia**	18	0.24	10.59		2.0
**Hypertension**	0	0.00	0.00		
**Epistaxis**	1	0.01	0.59		0.5
**Major**	3	0.04		0.01 (0.01-0.9)	
**Aspiration** [2]	1	0.01			
**Shock/Reanimation**	2	0.03			
**Mortality**	2	0.03		0.004 (0.0006-0.5) [3]	

Abbreviations: AE: adverse events.

[1] Comparison with reference study [1]. In parentheses, results of all four studies with >100000 patients [2-5].

[2] Rated as a mild complication in the reference study [1].

[3] [2-7]

1. Behrens, A., A. Kreuzmayr, H. Manner, H. Koop, A. Lorenz, C. Schaefer, M. Plauth, J. U. Jetschmann, C. von Tirpitz, M. Ewald, M. Sackmann, W. Renner, M. Kruger, D. Schwab, W. Hoffmann, O. Engelke, O. Pech, F. Kullmann, S. Pampuch, B. Lenfers, U. Weickert, D. Schilling, S. Boehm, S. Beckebaum, V. Cicinnati, J. F. Erckenbrecht, F. L. Dumoulin, C. Benz, T. Rabenstein, G. Haltern, M. Balsliemke, C. de Mas, G. Kleber, C. Pehl, C. Vogt, R. Kiesslich, W. Fischbach, I. Koop, J. Kuehne, M. Breidert, N. L. Sass, A. May, C. Friedrich, R. Veitt, R. Porschen, M. Ellrichmann, A. Arlt, W. Schmitt, M. Dollhopf, W. Schmidbaur, A. Dignass, V. Schmitz, J. Labenz, G. Kaiser, A. Krannich, N. Barteska, and C. Ell. "Acute Sedation-Associated Complications in Gi Endoscopy (Prosed 2 Study): Results from the Prospective Multicentre Electronic Registry of Sedation-Associated Complications." *Gut* 68, no. 3 (2019): 445-52.
2. Sharma, V. K., C. C. Nguyen, M. D. Crowell, D. A. Lieberman, P. de Garmo, and D. E. Fleischer. "A National Study of Cardiopulmonary Unplanned Events after Gi Endoscopy." *Gastrointest Endosc* 66, no. 1 (2007): 27-34.
3. Rex, D. K., V. P. Deenadayalu, E. Eid, T. F. Imperiale, J. A. Walker, K. Sandhu, A. C. Clarke, L. C. Hillman, A. Horiuchi, L. B. Cohen, L. T. Heuss, S. Peter, C. Beglinger, J. A. Sinnott, T. Welton, M. Rofail, I. Subei, R. Sleven, P. Jordan, J. Goff, P. D. Gerstenberger, H. Munnings, M. Tagle, B. W. Sipe, T. Wehrmann, J. A. Di Palma, K. E. Occhipinti, E. Barbi, A. Riphaus, S. T. Amann, G. Tohda, T. McClellan, C. Thueson, J. Morse, and N. Meah. "Endoscopist-Directed Administration of Propofol: A Worldwide Safety Experience." *Gastroenterology* 137, no. 4 (2009): 1229-37; quiz 518-9.

4. Behrens, A., J. Labenz, A. Schuler, W. Schroder, M. Runzi, R. U. Steinmann, C. R. de Mas, A. Kreuzmayr, K. Barth, M. J. Bahr, E. Burmester, J. F. Erckenbrecht, T. Frieling, F. L. Dumoulin, B. Pfaffenbach, W. Schepp, A. Schneider, G. Kleber, M. Meiborg, S. Bohm, C. Dietrich, C. F. Dietrich, U. Gottschalk, and C. Ell. "[How Safe Is Sedation in Gastrointestinal Endoscopy? A Multicentre Analysis of 388,404 Endoscopies and Analysis of Data from Prospective Registries of Complications Managed by Members of the Working Group of Leading Hospital Gastroenterologists (Algk)]." *Z Gastroenterol* 51, no. 5 (2013): 432-6.
5. Frieling, T., J. Heise, C. Kreysel, R. Kuhlen, and M. Schepke. "Sedation-Associated Complications in Endoscopy--Prospective Multicentre Survey of 191142 Patients." *Z Gastroenterol* 51, no. 6 (2013): 568-72.
6. Arrowsmith, J. B., B. B. Gerstman, D. E. Fleischer, and S. B. Benjamin. "Results from the American Society for Gastrointestinal Endoscopy/U.S. Food and Drug Administration Collaborative Study on Complication Rates and Drug Use During Gastrointestinal Endoscopy." *Gastrointest Endosc* 37, no. 4 (1991): 421-7.
7. Quine, M. A., G. D. Bell, R. F. McCloy, J. E. Charlton, H. B. Devlin, and A. Hopkins. "Prospective Audit of Upper Gastrointestinal Endoscopy in Two Regions of England: Safety, Staffing, and Sedation Methods." *Gut* 36, no. 3 (1995): 462-7.
